# Supplementary material for: Structural insight into an Arl1–ArfGEF complex involved in Golgi recruitment of a GRIP-domain golgin
Source: Nat Commun. 2024 Mar 2;15:1942. doi: 10.1038/s41467-024-46304-w (PMC10908827; doi:10.1038/s41467-024-46304-w)

Supplementary Information for

Structural insight into an Arl1–ArfGEF complex involved in Golgi recruitment of a GRIP-domain golgin

By Duan et al.

This file contains

2 Supplementary Tables

14 Supplementary Figures

Supplementary Table 1. Cryo-EM data collection, refinement, and validation statistics

	Arl1–Gea2 complex (EMD-28743, PDB 8EZJ)			Gea2 (EMDB-28748, PDB 8EZQ)		
	Consensus	Gea2 protomer A HDS local	Gea2 protomer B HDS local	Consensus	Gea2 protomer A HDS local	Gea2 protomer B HDS local
**Data collection and processing**						
Magnification	105,000	105,000	105,000	105,000	105,000	105,000
Voltage (kV)	300	300	300	300	300	300
Electron dose (e^-/Å^2)	69	69	69	69	69	69
Defocus (-μm)	1.3-1.7	1.3-1.7	1.3-1.7	1.3-1.7	1.3-1.7	1.3-1.7
Pixel size (Å)	0.828	0.828	0.828	0.828	0.828	0.828
Symmetry	C1	C1	C1	C1	C1	C1
Initial particle images (no.)	1,703,885	1,703,885	1,703,885	2,450,762	2,450,762	2,450,762
Final particle images (no.)	712,548	712,548	712,548	435,876	435,876	435,876
Map resolution (Å)	3.3	3.8	3.8	3.7	4.1	4.2
FSC threshold	0.143	0.143	0.143	0.143	0.143	0.143
Map resolution range (Å)	1.8-9.7	2.2-7.6	2.4-8.9	2.2-11	2.5-6.6	2.2-6.5
**Refinement**						
Initial model	AF-P39993-F1-model_v3, PDB 5EE5			Arl1–Gea2 complex of this study		
Model resolution (Å)	3.2			4.0		
FSC threshold	0.5			0.5		
Map sharpening *B* factor (Å^2)	-127.7	-159.6	-159.3	-136.1	-203.9	-198.6
Model composition						
Non-hydrogen atoms	23,215			20,456		
Protein residues	2,891			2,545		
Ligands	2			0		
B factors (Å^2)						
Protein	83.18			109.07		
Ligand	140.10			–		
R.m.s. deviations						
Bond lengths (Å)	0.004			0.003		
Bond angles (°)	0.554			0.613		
**Validation**						
MolProbity score	2.06			2.29		
Clashscore	10.74			17.77		
Poor rotamers (%)	0.23			0.42		
Ramachandran plot						
Favored (%)	91.44			90.40		
Allowed (%)	8.56			9.60		
Disallowed (%)	0.00			0.00		

Supplementary Table 2. **Yeast strains and plasmids used in the study**

Strain	Genotype	Plasmid	Source
CJY092	MATα *ura3-52 leu2-Δ1 his3-Δ200 lys2-801 ade2-101 trp1-Δ63 gea1::HIS3 gea2::HIS3*	pSKP1 CEN-URA3-GEA2	Chantalat et al., 2004
BJ701	CJY092	pRS315-ADH1-Gea2-3xFLAG	This Study
BJ702	CJY092	pRS315	This Study
BJ703	CJY092	pRS315-ADH1-Gea2 P71A-3xFLAG	This Study
BJ704	CJY092	pRS315-ADH1-Gea2 L79A-3xFLAG	This Study
BJ705	CJY092	pRS315-ADH1-Gea2 N84A-3xFLAG	This Study
BJ706	CJY092	pRS315-ADH1-Gea2 P100A-3xFLAG	This Study
BJ707	CJY092	pRS315-ADH1-Gea2 K124A-3xFLAG	This Study
BJ708	CJY092	pRS315-ADH1-Gea2 L128A-3xFLAG	This Study
BJ709	CJY092	pRS315-ADH1-Gea2 L167A-3xFLAG	This Study
BJ710	CJY092	pRS315-ADH1-Gea2 F171A-3xFLAG	This Study
BJ711	CJY092	pRS315-ADH1-Gea2 L167F-3xFLAG	This Study
BJ712	CJY092	pRS315-ADH1-Gea2 E368K-3xFLAG	This Study
BJ713	CJY092	pRS315-ADH1-Gea2 E460R-3xFLAG	This Study
BJ714	CJY092	pRS315-ADH1-Gea2 E464R-3xFLAG	This Study
BJ715	CJY092	pRS315-ADH1-Gea2 R472E-3xFLAG	This Study
BJ716	CJY092	pRS315-ADH1-Gea2 K124A, L128A-3xFLAG	This Study
BJ717	CJY092	pRS315-ADH1-Gea2 L167A, F171A-3xFLAG	This Study
BJ718	CJY092	pRS315-ADH1-Gea2 DCBΔ-3xFLAG	This Study
BY4741 *gea2Δ*	MATa *his3, leu2, met15, ura3, gea2::KanMX6*		Tsai et al., 2013
BJ722	BY4741 *gea2Δ*	pRS315-ADH1-Gea2-3xFLAG, pRS416	This Study
BJ724	BY4741 *gea2Δ*	pRS315-ADH1-Gea2-3xFLAG, pRS416-ADH1-Gea2-3xHA	This Study

BJ725	BY4741 *gea2∆*	pRS315-ADH1-Gea2 DCB∆-3xFLAG, pRS416-ADH1-Gea2-3xHA	This Study
BJ726	BY4741 *gea2∆*	pRS315-ADH1-Gea2 K124A, L128A-3xFLAG, pRS416-ADH1-Gea2-3xHA	This Study
BJ727	BY4741 *gea2∆*	pRS315-ADH1-Gea2 L167A, F171A-3xFLAG, pRS416-ADH1-Gea2-3xHA	This Study
BJ728	BY4741 *gea2∆* Sec7-GFP::URA3	pVT101U-ADH1-mCherry-IMH1	This Study
BJ729	BJ728	pRS315-ADH1-Gea2-3xFLAG	This Study
BJ730	BJ728	pRS315	This Study
BJ744	BJ728	pRS315-ADH1-Gea2 K124A, L128A-3xFLAG	This Study
BJ745	BJ728	pRS315-ADH1-Gea2 L167A, F171A-3xFLAG	This Study
BJ746	BJ728	pRS315-ADH1-Gea2 DCB∆-3xFLAG	This Study
BY4741 *gea2/arl1∆*	MATa *his3, leu2, met15, ura3, gea2::KanMX6, arl1*		Tsai et al., 2013
BJ750	BY4741 *gea2/arl1∆*	pRS315-ADH1-Gea2-3xFLAG, pRS416-TEF1-Arl1 L69A-3xHA	This Study
BJ751	BY4741 *gea2/arl1∆*	pRS315-ADH1-Gea2-3xFLAG, pRS416-TEF1-Arl1 Y78L-3xHA	This Study
BJ752	BY4741 *gea2/arl1∆*	pRS315-ADH1-Gea2-3xFLAG, pRS416-TEF1-Arl1 C81A-3xHA	This Study
BJ753	BY4741 *gea2/arl1∆*	pRS315-ADH1-Gea2 DCB∆-3xFLAG, pRS416-TEF1-Arl1-3xHA	This Study
BY4741 *arl1∆*	MATa *his3, leu2, met15, ura3, arl1::KanMX6*		Tsai et al., 2013
BJ755	BY4741 *arl1∆* Sec7-GFP::URA3	pVT101U-ADH1-mCherry-IMH1	This Study
BJ756	BJ755	pRS313	This Study
BJ757	BJ755	pRS313-Arl1	This Study
BJ758	BJ755	pRS313-Arl1 L69A	This Study
BJ759	BJ755	pRS313-Arl1 Y78L	This Study
BJ760	BJ755	pRS313-Arl1 C81A	This Study
BJ761	BY4741 *arl1∆* Sec7-mCherry::URA3	pRS416-Arl1-mNG	This Study

BJ762	BY4741 *arl1*Δ Sec7-mCherry::URA3	pRS416-Arl1 L69A-mNG	This Study
BJ763	BY4741 *arl1*Δ Sec7-mCherry::URA3	pRS416-Arl1 Y78L-mNG	This Study
BJ764	BY4741 *gea2*Δ Sec7-mCherry::URA3	pRS315-ADH1-Gea2-mNG	This Study
BJ765	BY4741 *gea2*Δ Sec7-mCherry::URA3	pRS315-ADH1-Gea2 DCBΔ-mNG	This Study
BJ766	BY4741 *gea2*Δ Sec7-mCherry::URA3	pRS315-ADH1-Gea2 K124A, L128A-mNG	This Study
BJ767	BY4741 *gea2*Δ Sec7-mCherry::URA3	pRS315-ADH1-Gea2 L167A, F171A-mNG	This Study
BJ769	BY4741 *gea2/arl1*Δ	pRS315-ADH1-Gea2-3xFLAG, pRS416-TEF1-Arl1-3xHA	This Study
BJ770	BY4741 *arl1*Δ Sec7-mCherry::URA3	pRS416-TEF1-Arl1 C81A-mNG	This Study
BJ771	BY4741 *gea2/arl1*Δ	pRS315-ADH1-Gea2 P71A-3xFLAG, pRS416-TEF1-Arl1-3xHA	This Study
BJ772	BY4741 *gea2/arl1*Δ	pRS315-ADH1-Gea2 L79A-3xFLAG, pRS416-TEF1-Arl1-3xHA	This Study
BJ773	BY4741 *gea2/arl1*Δ	pRS315-ADH1-Gea2 P100A-3xFLAG, pRS416-TEF1-Arl1-3xHA	This Study
BJ774	BY4741 *gea2/arl1*Δ	pRS315-ADH1-Gea2-3xFLAG, pRS416-TEF1-Arl1 L69A, Y78L-3xHA	This Study
BJ775	BJ755	pRS313-Arl1 L69A, Y78L	This Study
BJ776	BY4741 *arl1*Δ Sec7-mCherry::URA3	pRS416-Arl1 L69A, Y78L-mNG	This Study

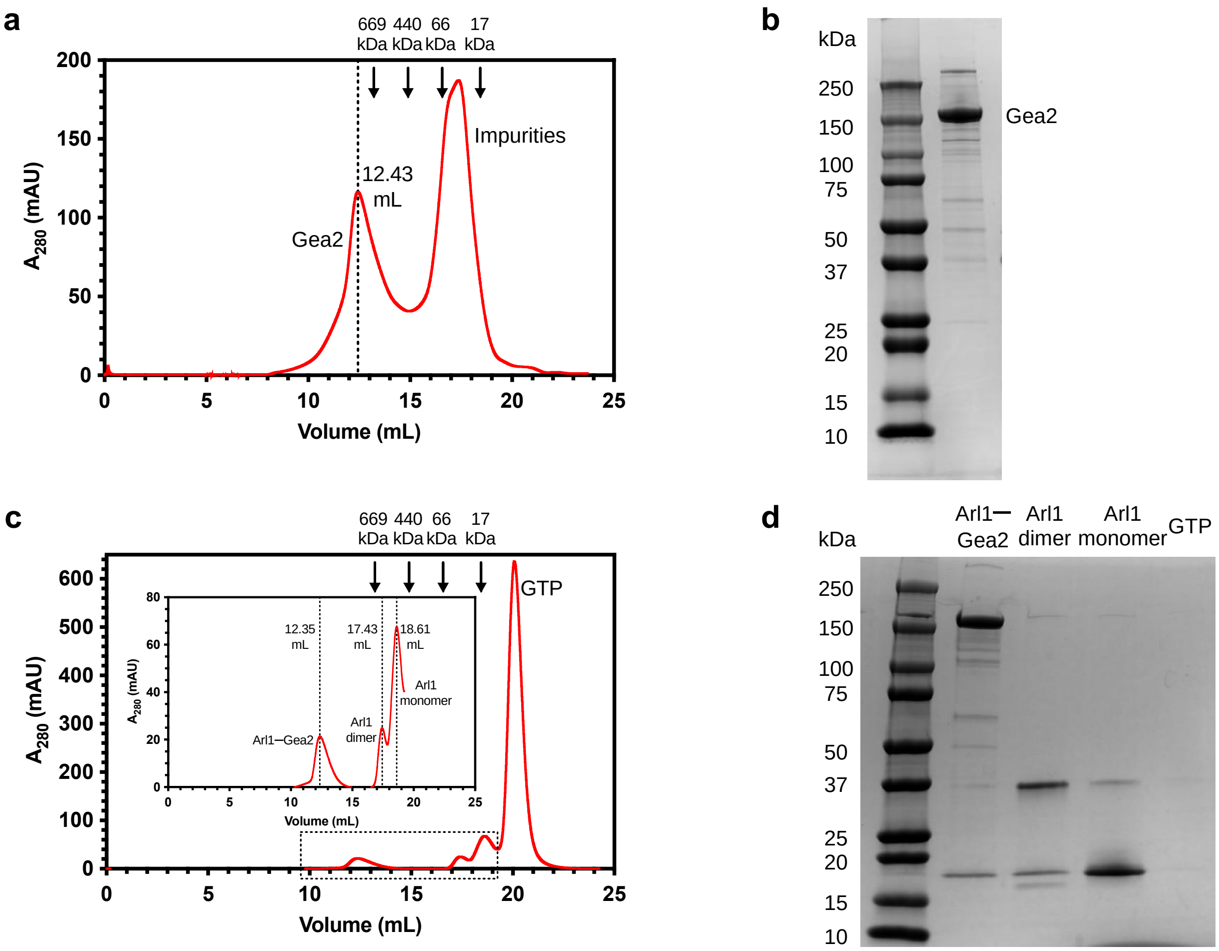

Supplementary Figure 1. Purification of the full-length *S. cerevisiae* Gea2 and *in vitro* binding analysis of Gea2 with Arl1. a) Gel filtration profile of Gea2. The Gea2 dimer had a calculated mass of 337 kDa but was eluted at 12.43 mL, corresponding to mass larger than the 669 kDa standard, due to its highly extended shape. **b)** Coomassie blue-stained SDS-PAGE gel of the Gea2 peak fraction. **c)** Gel filtration profile of the mixture of Arl1 and Gea2. Inset is an enlarged view of the profile in ~10 – 19 ml elution volume, showing peaks for the Arl1–Gea2 complex and as well as excess Arl1. **d)** Coomassie blue-stained SDS-PAGE gel of elution peak fractions corresponding to the Arl1–Gea2 complex, the Alr1 dimer, the Arl1 monomer, and the free GTP. Source data are provided as a Source Data file.

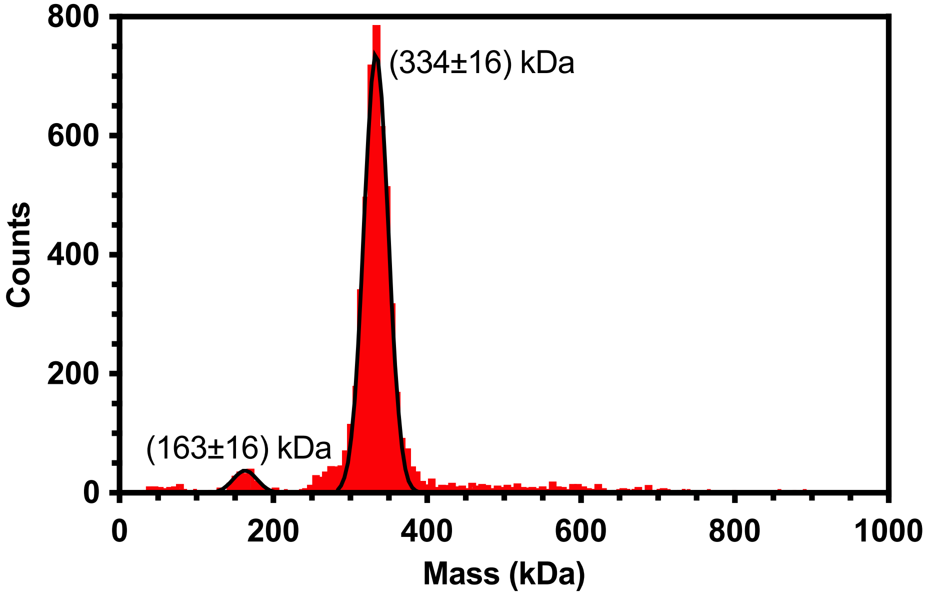

Supplementary Figure 2. Mass photometry analysis of purified Gea2 in solution. The histogram shows population distributions of Gea2 particles in solution. The predominant peak at 334 kDa represents dimers and the minor peak at 163 kDa represents monomers. The peaks were fitted by Gaussian distribution to derive the average molecular masses and standard deviations. Source data are provided as a Source Data file.

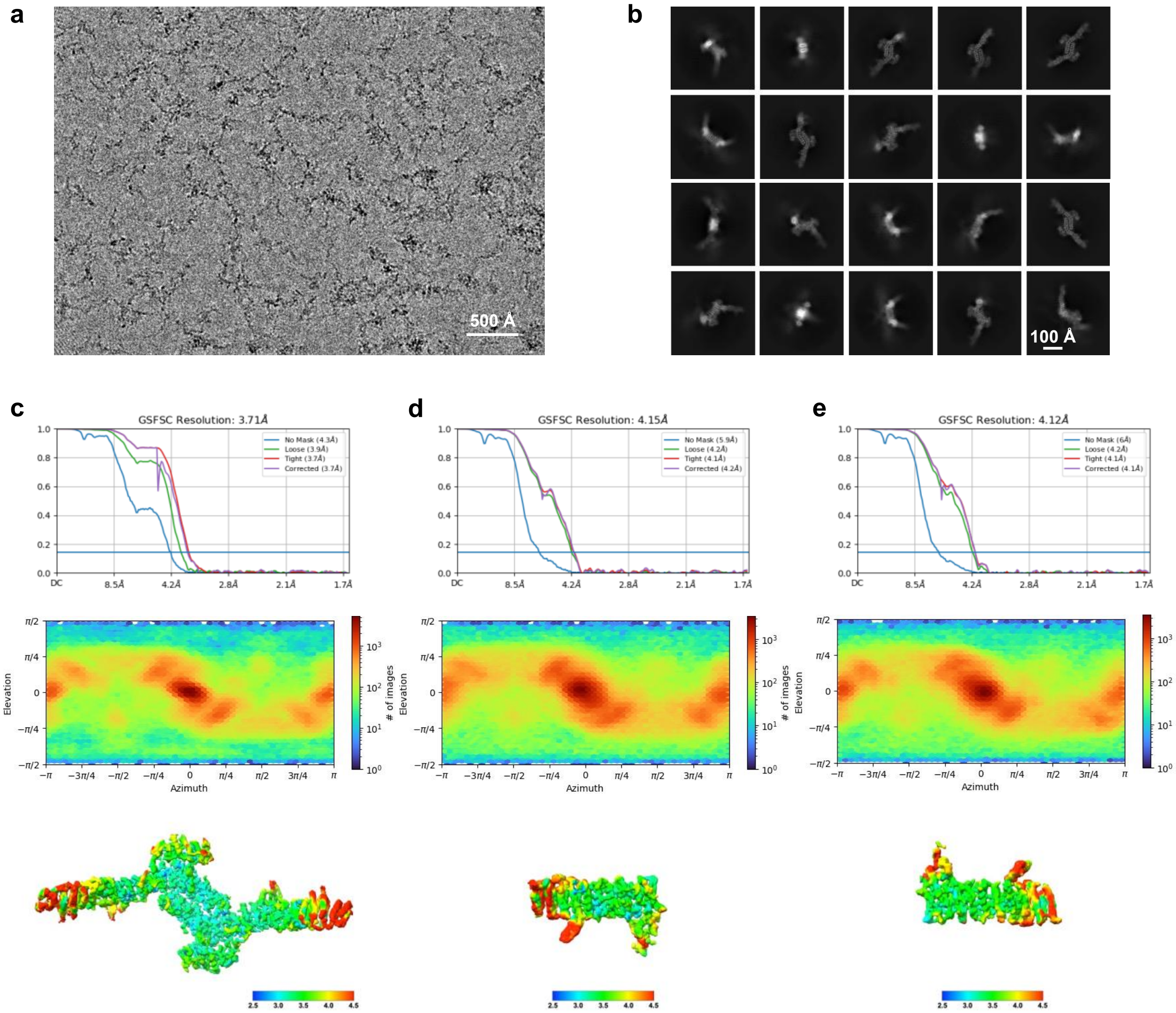

Supplementary Figure 3. Cryo-EM reconstruction of the Gea2 dimer and resolution estimation of the 3D EM maps. Representative raw micrograph selected from a total of 13,636 micrographs. (**a**) and 2D classes (**b**). The gold-standard Fourier shell correlation (GSFSC) curve for the 3D reconstruction (top), angular distribution heat map (middle) and local resolution map (bottom) for the consensus reconstruction (**c**), Gea2 protomer B HDS local refinement map (**d**) and Gea2 protomer A HDS local refinement map (**e**).

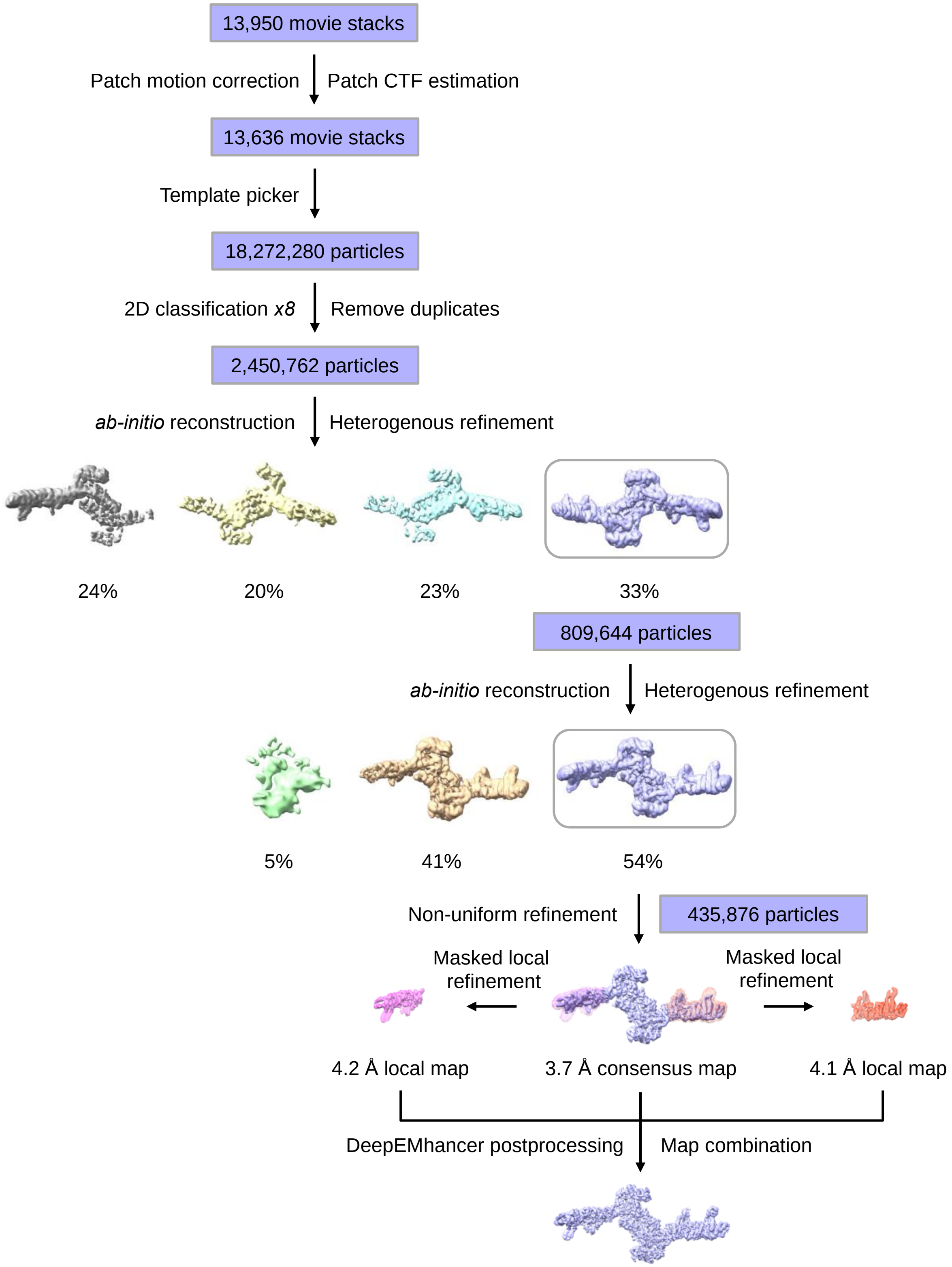

Supplementary Figure 4. Flowchart of cryo-EM data processing for the Gea2 dimer.

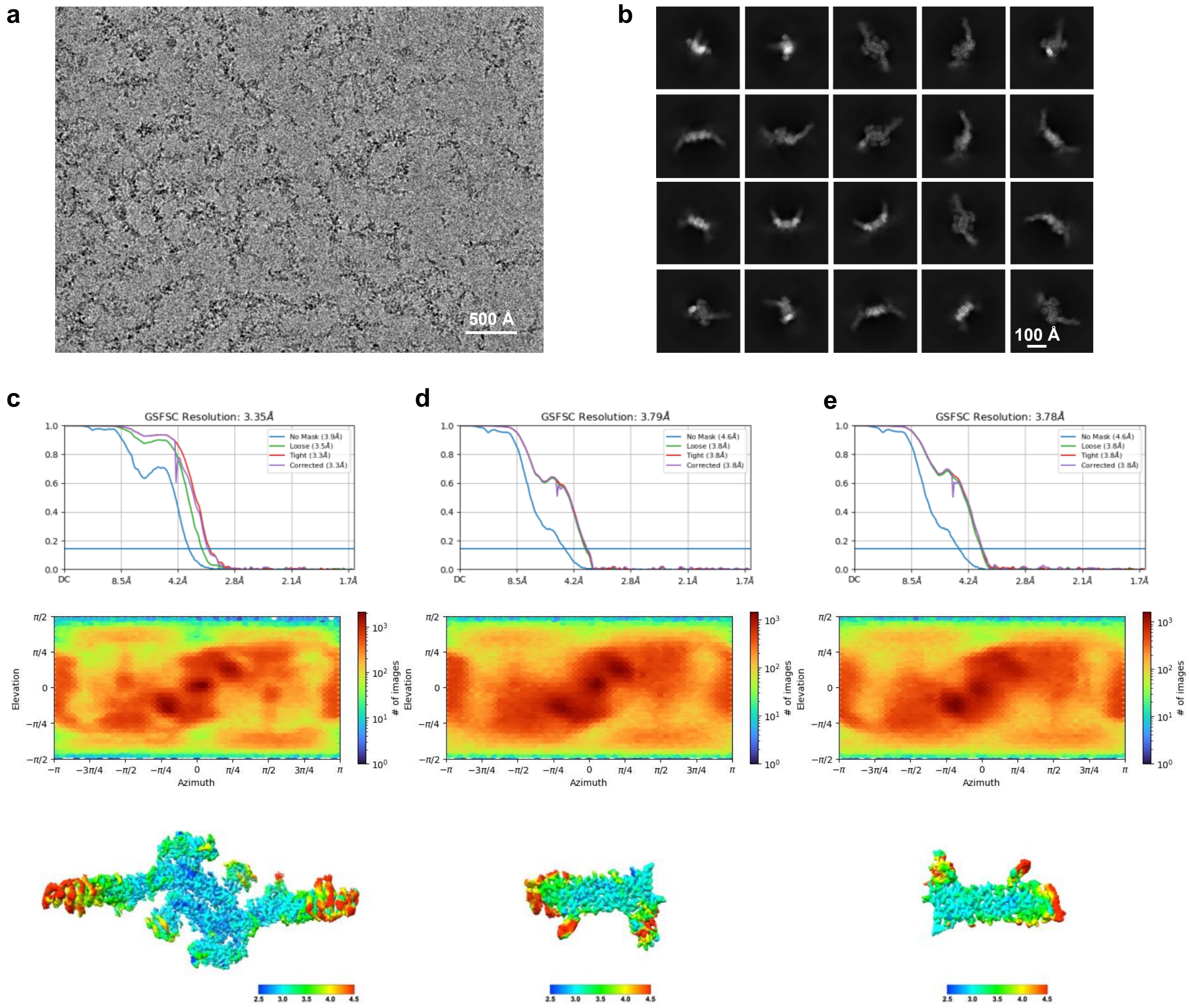

Supplementary Figure 5. Cryo-EM reconstruction of the Arl1–Gea2 complex and resolution estimation of 3D EM maps. Representative raw micrograph selected from a total of 20,027 micrographs. (**a**) and 2D classes (**b**). The gold-standard Fourier shell correlation (GSFSC) curve for the 3D reconstruction (top), angular distribution heat map (middle) and local resolution map (bottom) for the consensus reconstruction (**c**), Gea2 protomer B HDS local refinement map (**d**) and Gea2 protomer A HDS local refinement map (**e**).

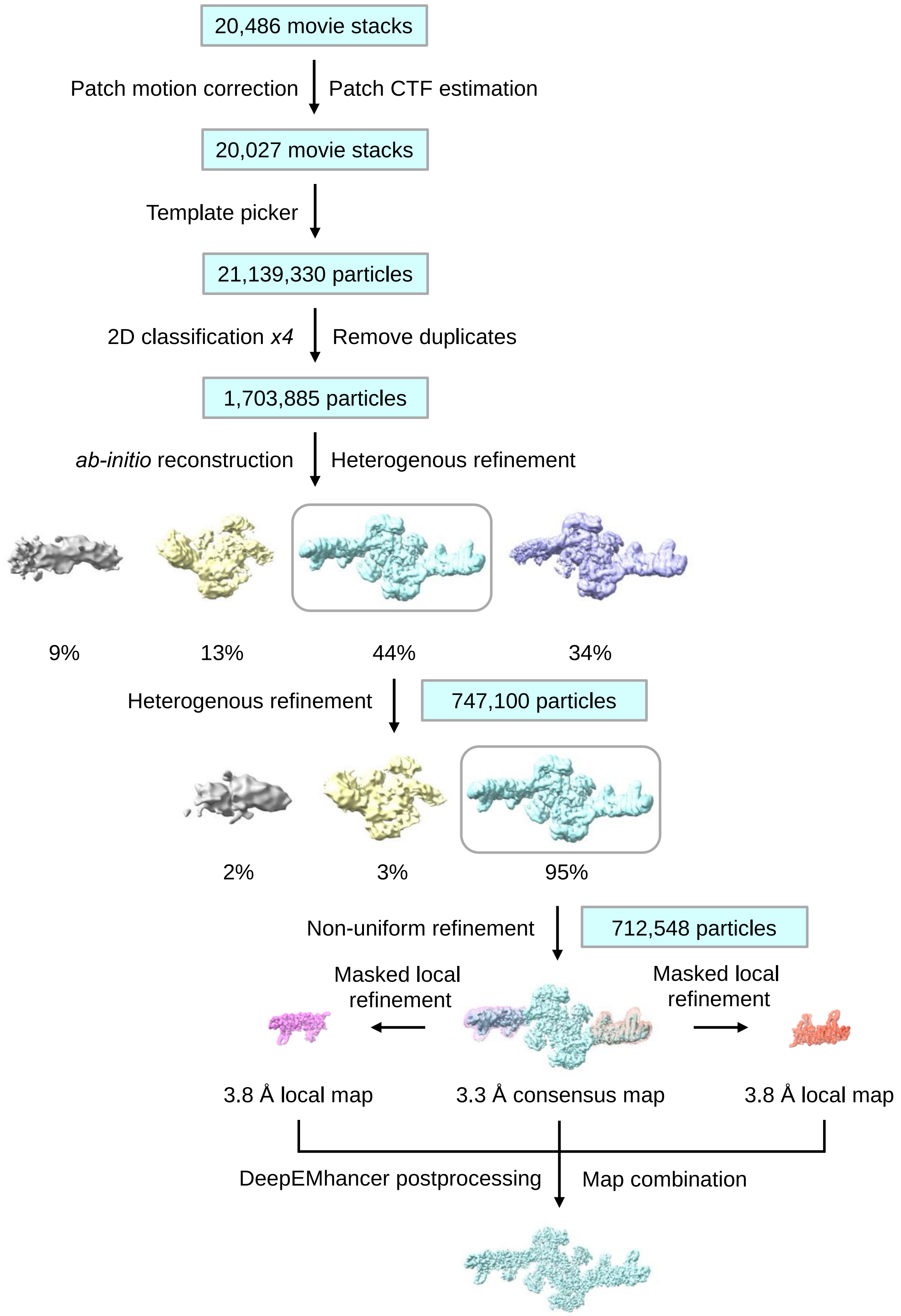

Supplementary Figure 6. Flowchart of cryo-EM data processing for the Arl1–Gea2 complex.

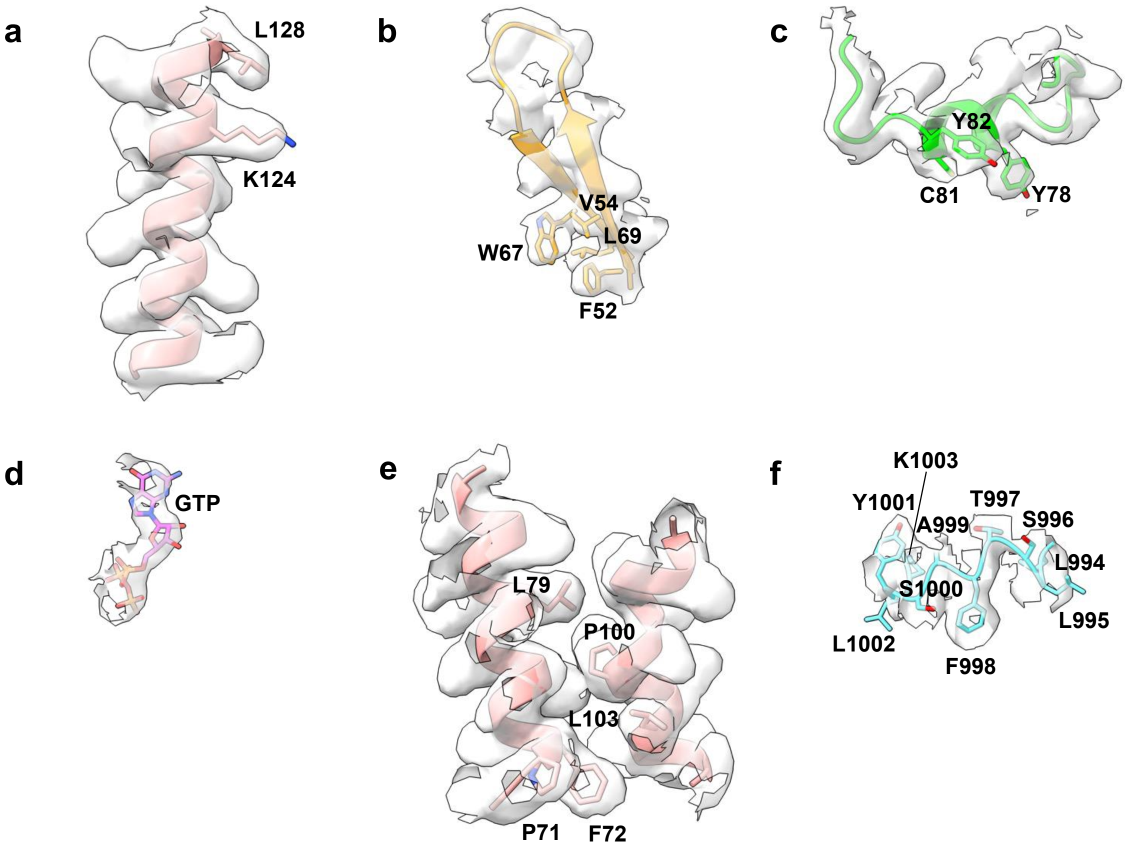

Supplementary Figure 7. Superimposition of EM density map and atomic model of key structural elements. a) The DCB domain helix carrying K124 and L128 for Gea2 dimerization. **b)** Interswitch hairpin of Arl1 carrying F52, V54, W67 and L69 for interaction with Gea2. **c)** Switch 2 of Arl1 carrying Y78, Y82 and C81 for interaction with Gea2. **d)** GTP in Arl1. **e)** The DCB domain helices of Gea2 carrying P71, F72, L79, P100 and L103 for interaction with Arl1. **f)** Heal-like motif of Gea2 for interaction with the membrane. Key residues and cofactors are shown in sticks, EM densities are shown in transparent surfaces.

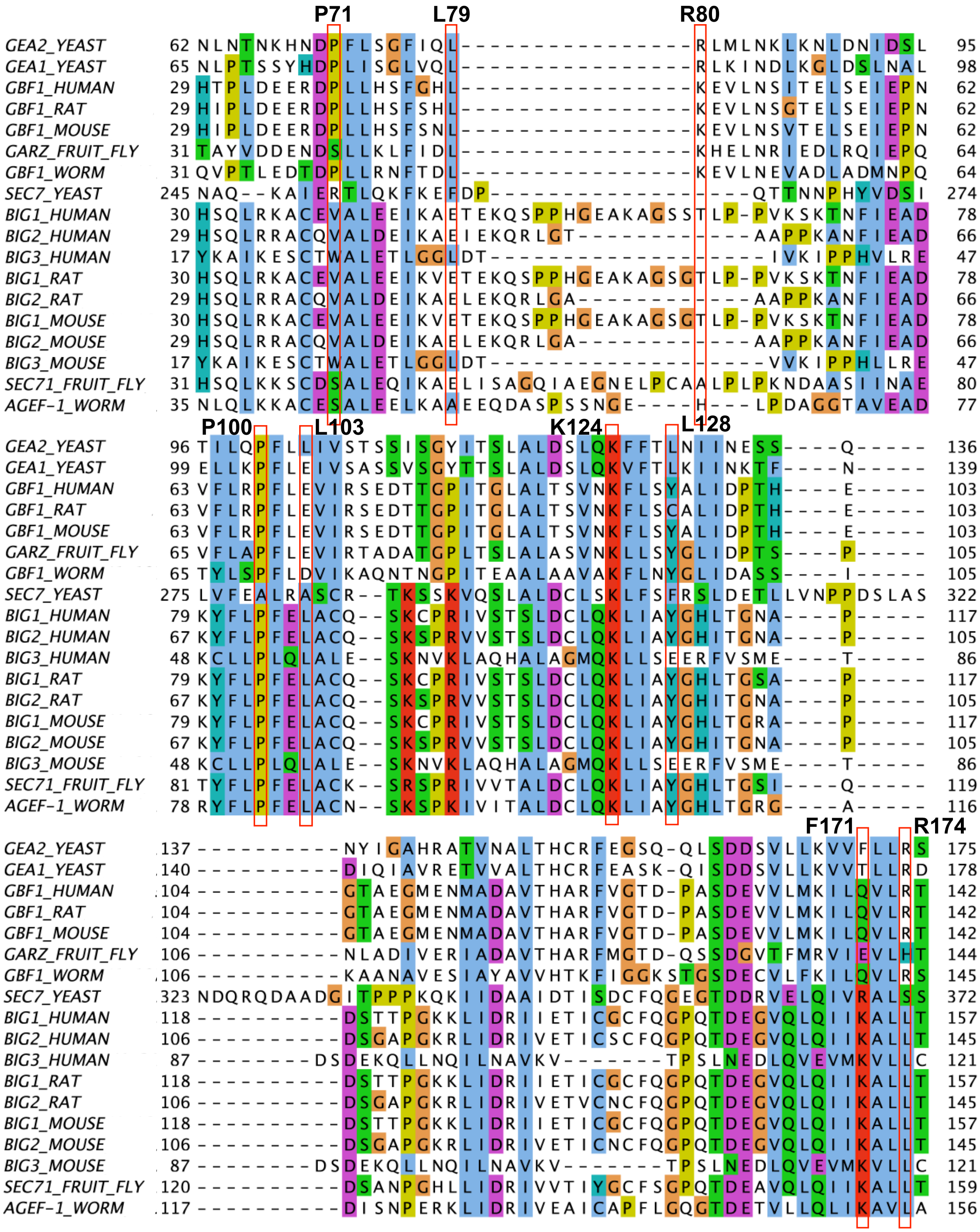

Supplementary Figure 8. Sequence alignment of the Gea2 DCB domains. Yeast: *Saccharomyces cerevisiae*; Human: *Homo sapiens*; Rat: *Rattus norvegicus*; Mouse: *Mus musculus*; Fruit fly: *Drosophila melanogaster*; Worm: *Caenorhabditis elegans*.

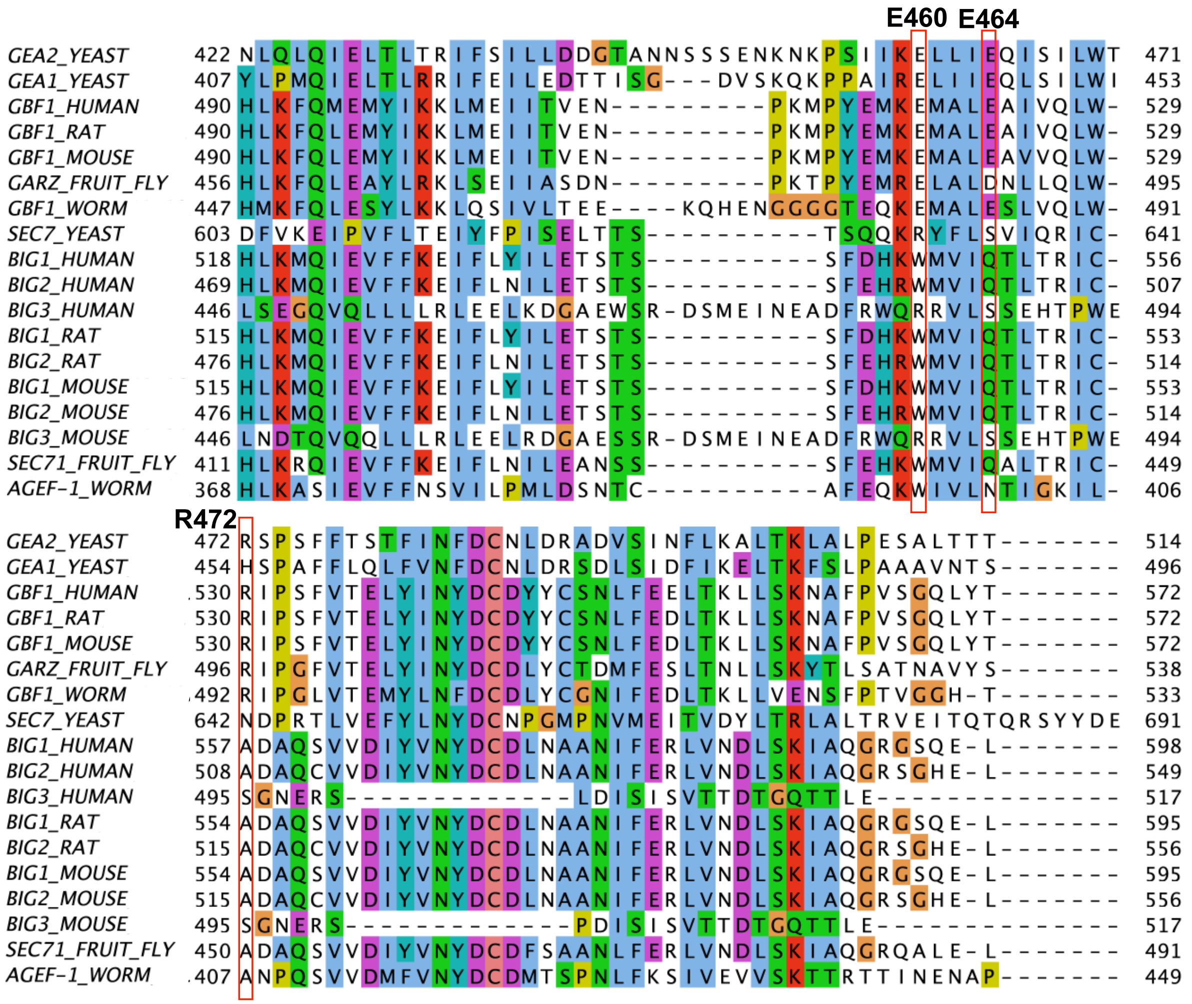

Supplementary Figure 9. Sequence alignment of the Gea2 HUS domains. Yeast: *Saccharomyces cerevisiae*; Human: *Homo sapiens*; Rat: *Rattus norvegicus*; Mouse: *Mus musculus*; Fruit fly: *Drosophila melanogaster*; Worm: *Caenorhabditis elegans*.

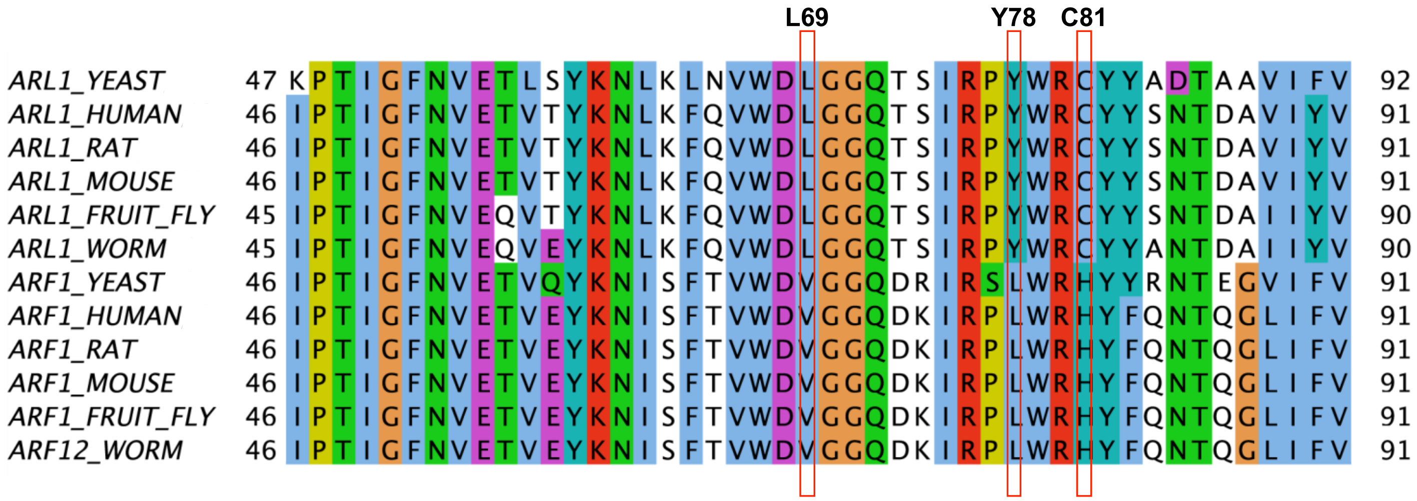

Supplementary Figure 10. Sequence alignment of the Arl1 Interswitch hairpin and Switch 2 regions. Yeast: *Saccharomyces cerevisiae*; Human: *Homo sapiens*; Rat: *Rattus norvegicus*; Mouse: *Mus musculus*; Fruit fly: *Drosophila melanogaster*; Worm: *Caenorhabditis elegans*.

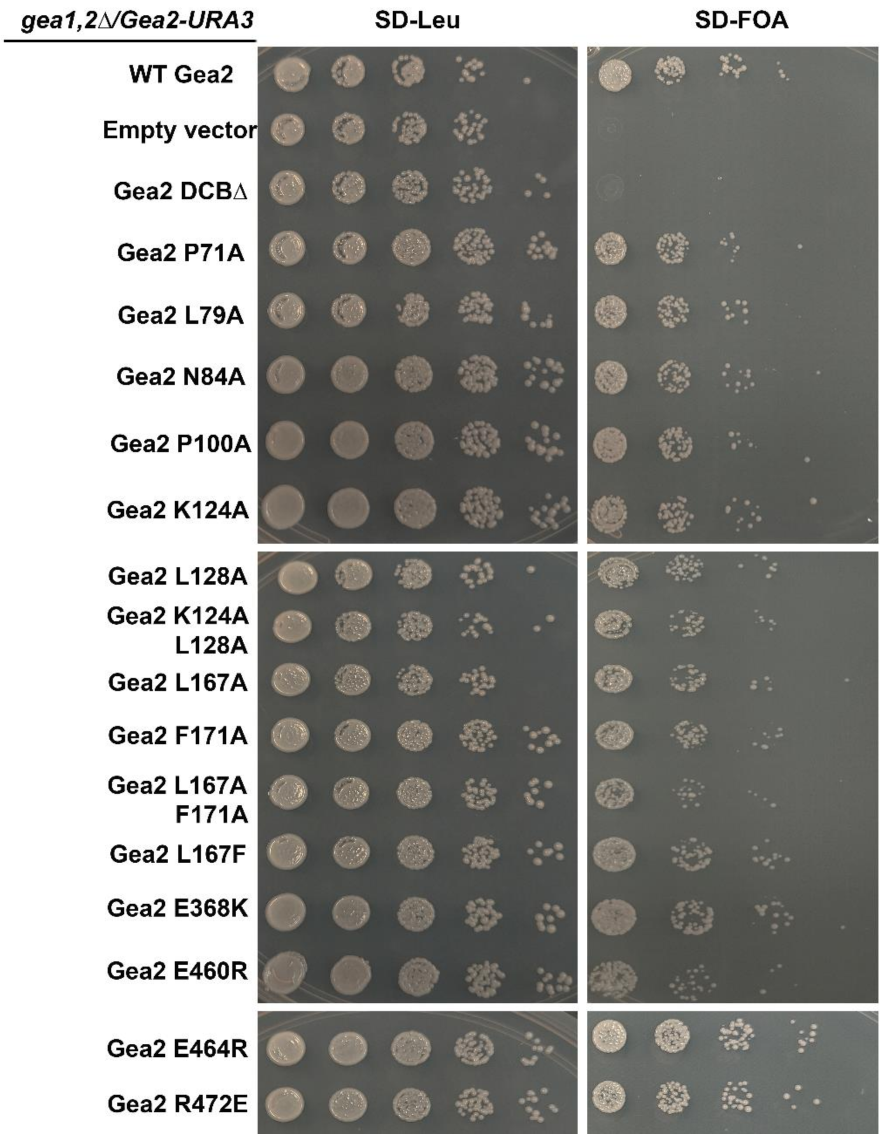

Supplementary Figure 11. All Gea2 mutants except Gea2 DCB∆ supported yeast cell viability. The *gea1∆gea2∆* pURA3-GEA2 strain was transformed with pRS415-Gea2 plasmids harboring the Gea2 mutants. Strains on the SD-Leu medium expressed both WT and mutant Gea2, but SD + 5-FOA medium allows expression of only Gea2 mutants.

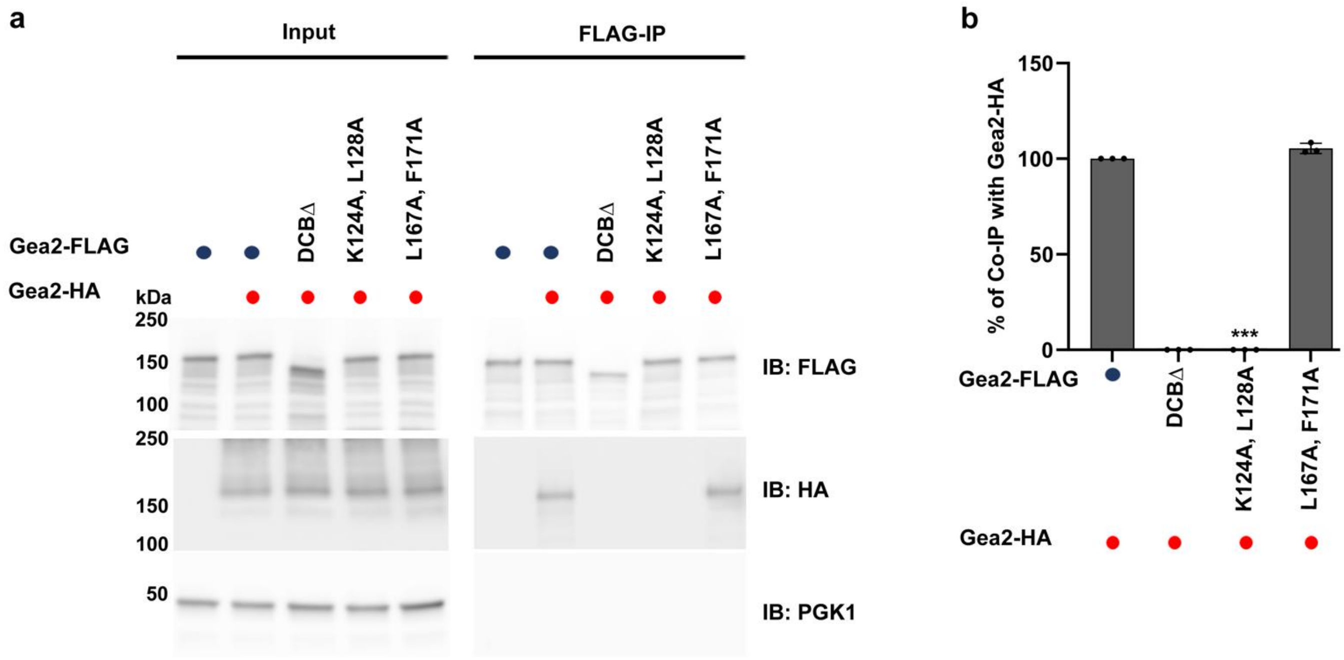

Supplementary Figure 12. Co-immunoprecipitation analysis to test the Gea2 dimerization interface. a) Gea2-3xFLAG tagged Gea2 dimer interface mutants were co-expressed with 3xHA tagged wild-type Gea2 in *gea2*Δ cells. Gea2-3xFLAG was immunoprecipitated by anti-FLAG magnetic beads, and the coprecipitated Gea2-3xHA was detected with anti-HA antibody. **b)** Quantification of the amount of coprecipitated Gea2-3xHA. Comparisons were calculated via a One-Way ANOVA followed by Dunnett's multiple comparisons test. n=3 represents data from 3 independent biological replicates. Error bars represent SD. *** indicates p value <0.001. Source data are provided as a Source Data file.

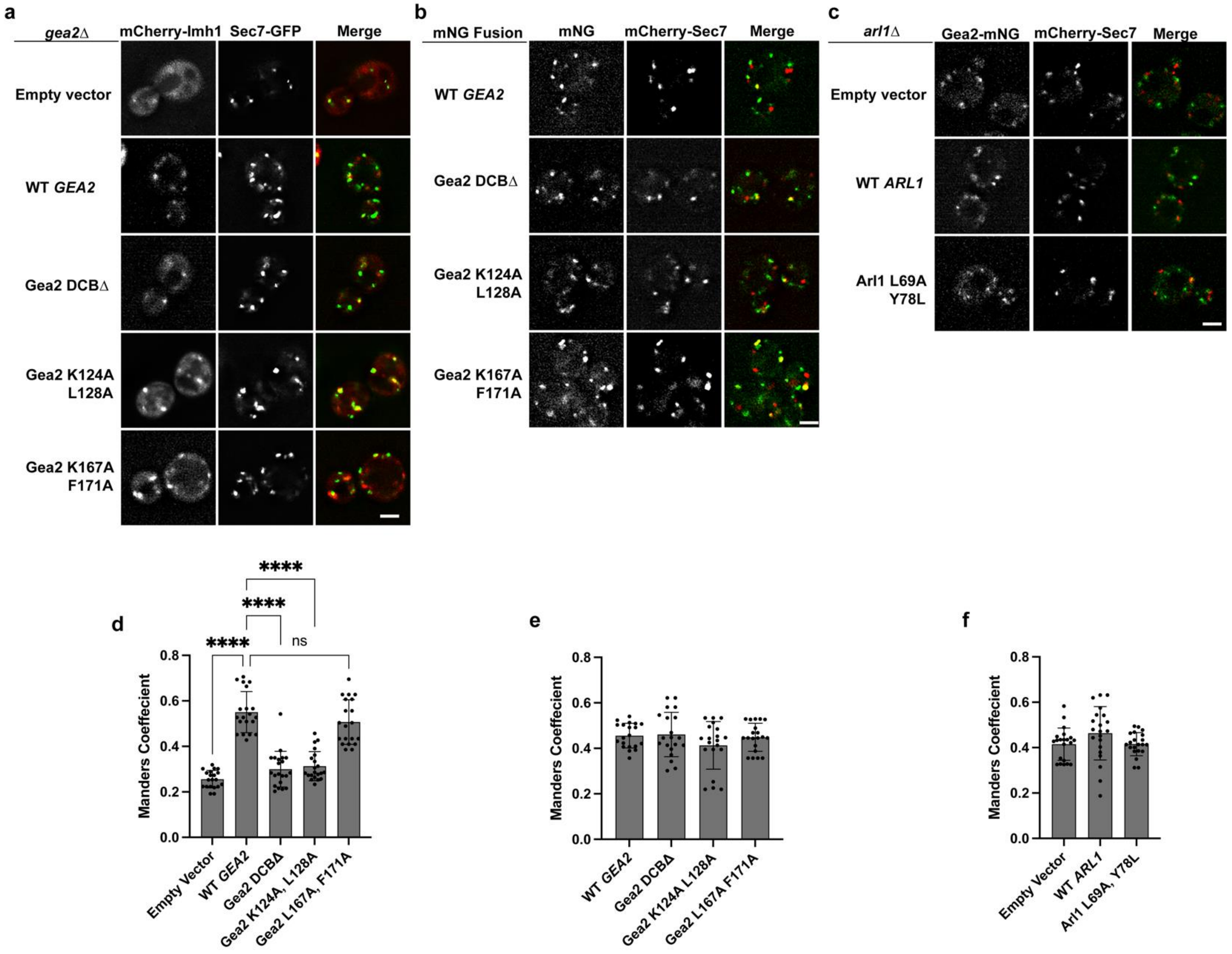

Supplementary Figure 13. Dimerization of Gea2 is required for the Golgi targeting of Imh1, while localization of Gea2 is not affected by its oligomeric states or its partner protein Arl1. a) Localizations of mCherry tagged Imh1 were examined in *gea2*Δ cells expressing either empty vector, WT Gea2 or Gea2 mutant variants. **b)** Localization of the mNeonGreen (mNG) tagged WT Gea2 and Gea2 dimer interface mutants. **c**) Localization of mNG tagged WT Gea2 in *arl1*Δ cells expressing either empty vector, WT Arl1 or Arl1 L69A, Y78L. Scale bar is 2 μm. **d-e)** Quantification via Manders' Coefficient of the proportion of mCherry-Imh1 (d) or mNG tagged Gea2 (e) colocalized with a late Golgi marker Sec7. **f)** Quantification via Manders' Coefficient of the proportion of Gea2 with Sec7 in *arl1*Δ cells expressing no Arl1, WT Arl1 or Arl1 L69A, Y78L. For all quantifications, data from (n=20) cells from three independent experiments of biological replicates were obtained and analyzed. Comparisons were calculated via a One-Way ANOVA followed by Tukey's post hoc test. Error bars represent SD. Source data are provided as a Source Data file.

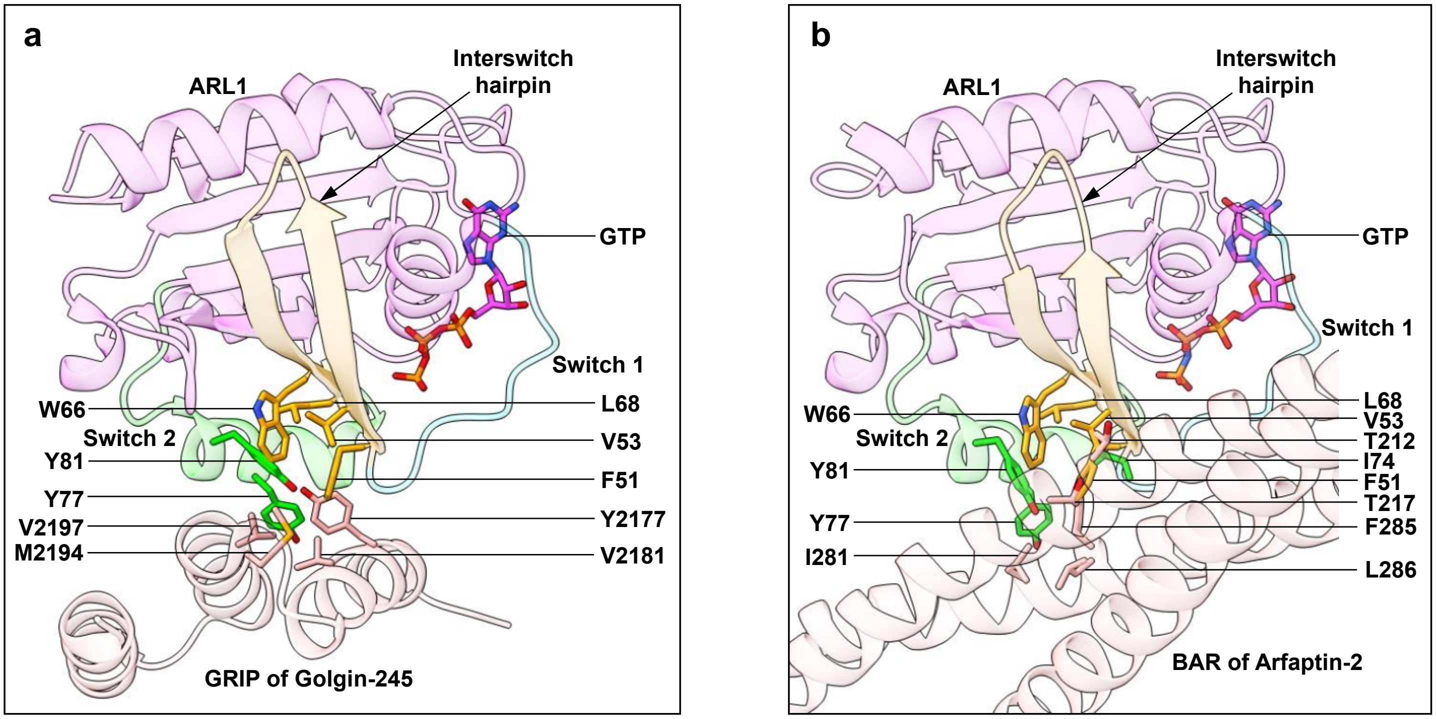

Supplementary Figure 14. The Arl1 interface for Gea2 binding is also used for interaction with its downstream effector proteins. a) Interface between ARL1 and Golgin-245 GRIP domain (PDB ID 1UPT). **b**) Interface between ARL1 and Arfaptin-2 BAR domain (PDB ID 4DCN).

Source Data for Supplementary Figures

Supplementary Figure 1b

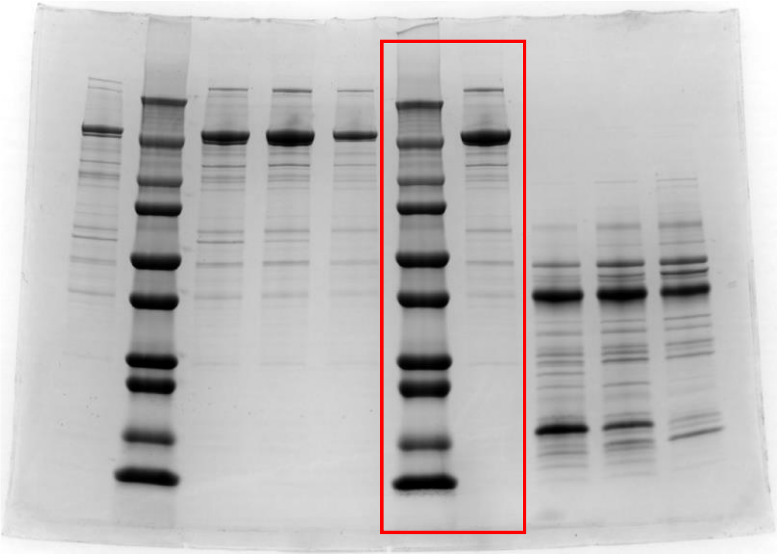

Supplementary Figure 1d

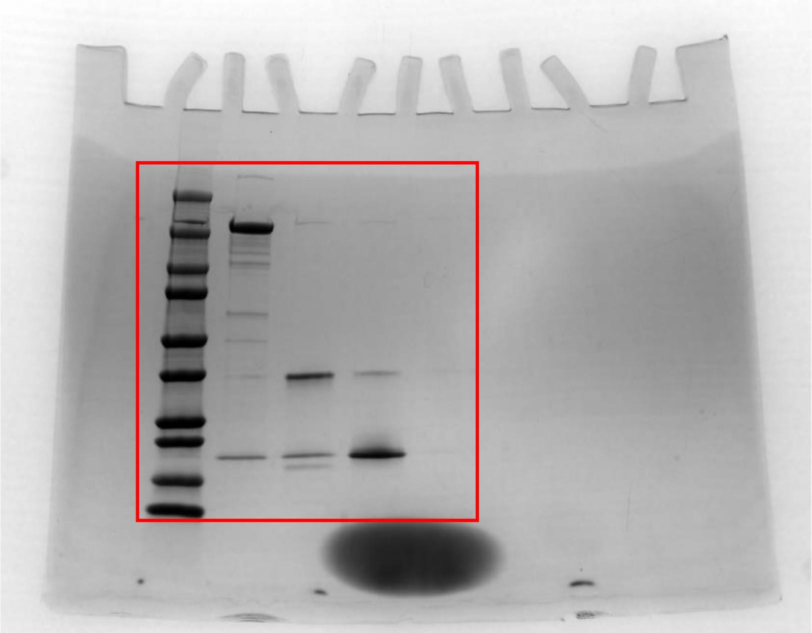

Supplementary Figure 11a

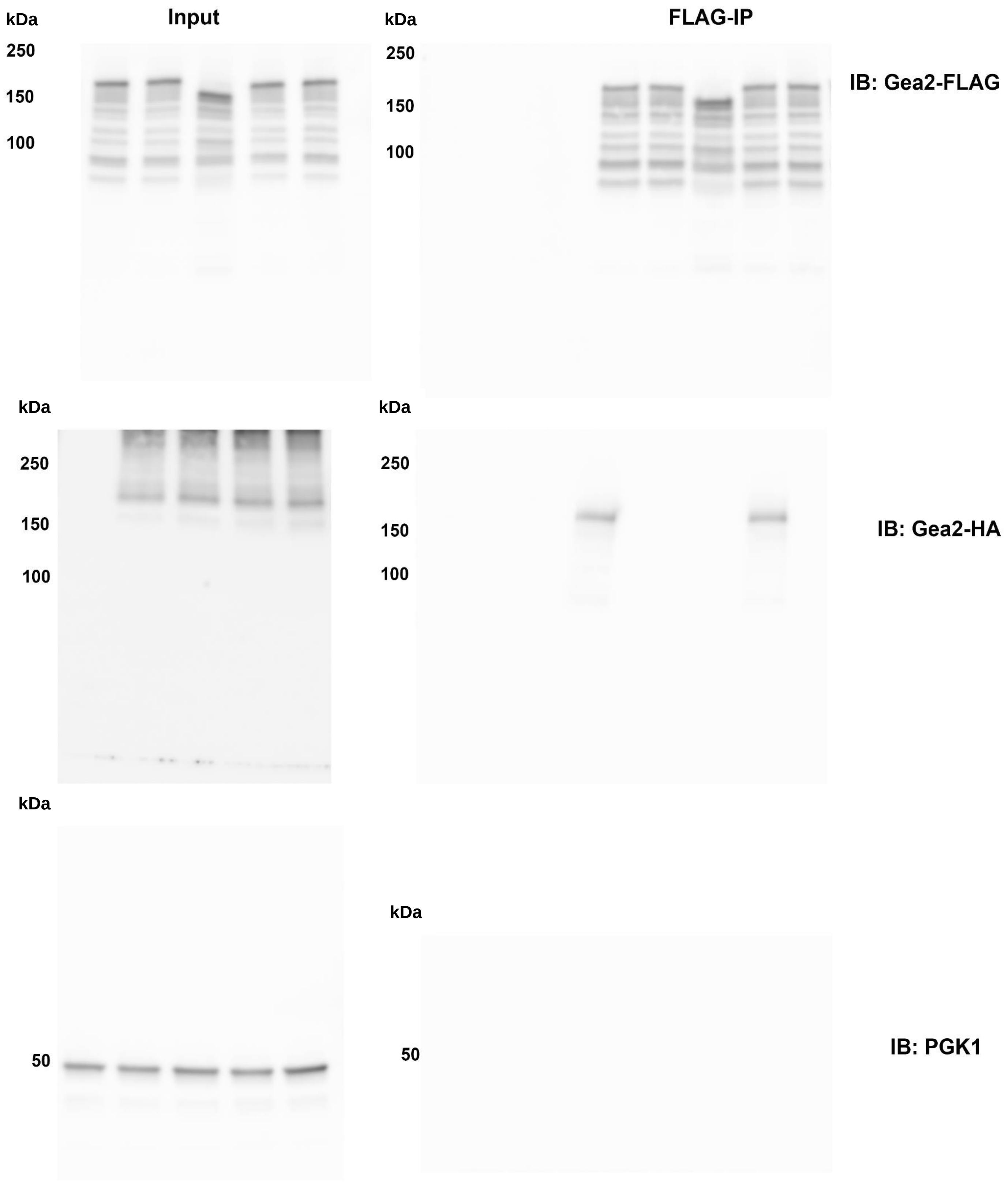

Supplement: Supplementary file 1 — Supplementary Information [file 41467_2024_46304_MOESM1_ESM.pdf]
