# Supplementary material for: Structural insight into an Arl1–ArfGEF complex involved in Golgi recruitment of a GRIP-domain golgin
Source: Nat Commun. 2024 Mar 2;15:1942. doi: 10.1038/s41467-024-46304-w (PMC10908827; doi:10.1038/s41467-024-46304-w)
Supplement: Supplementary file 3 — Reporting Summary [file 41467_2024_46304_MOESM3_ESM.pdf]

nature portfolio

Corresponding author(s): Todd R. Graham, Huilin Li

Last updated by author(s): Jan 31, 2024

Reporting Summary

Nature Portfolio wishes to improve the reproducibility of the work that we publish. This form provides structure for consistency and transparency in reporting. For further information on Nature Portfolio policies, see our Editorial Policies and the Editorial Policy Checklist.

Statistics

For all statistical analyses, confirm that the following items are present in the figure legend, table legend, main text, or Methods section.

n/a	Confirmed	
☐	☒	The exact sample size (*n*) for each experimental group/condition, given as a discrete number and unit of measurement
☐	☒	A statement on whether measurements were taken from distinct samples or whether the same sample was measured repeatedly
☐	☒	The statistical test(s) used AND whether they are one- or two-sided<br>*Only common tests should be described solely by name; describe more complex techniques in the Methods section.*
☐	☒	A description of all covariates tested
☒	☐	A description of any assumptions or corrections, such as tests of normality and adjustment for multiple comparisons
☐	☒	A full description of the statistical parameters including central tendency (e.g. means) or other basic estimates (e.g. regression coefficient) AND variation (e.g. standard deviation) or associated estimates of uncertainty (e.g. confidence intervals)
☐	☒	For null hypothesis testing, the test statistic (e.g. *F*, *t*, *r*) with confidence intervals, effect sizes, degrees of freedom and *P* value noted<br>*Give P values as exact values whenever suitable.*
☒	☐	For Bayesian analysis, information on the choice of priors and Markov chain Monte Carlo settings
☒	☐	For hierarchical and complex designs, identification of the appropriate level for tests and full reporting of outcomes
☒	☐	Estimates of effect sizes (e.g. Cohen's *d*, Pearson's *r*), indicating how they were calculated

Our web collection on statistics for biologists contains articles on many of the points above.

Software and code

Policy information about availability of computer code

Data collection	Cryo-EM data were collected using SerialEM v4.1.12. Fluorescence images were acquired using DeltaVision Elite Imaging System (GE Healthcare Life Sciences, Pittsburgh, PA) equipped with a 100×, 1.4 NA oil immersion objective lens. The images were deconvoluted by softWoRx software (GE Healthcare Life Science, 7.0.0 release RC6).
Data analysis	cryoSPARC v3.3, UCSF Chimera v1.16, UCSF ChimeraX v1.4, DynDom (version 1.5), Coot (version 0.9.8.3), Phenix (version 1.20.1), MolProbity (version 4.5.1), ImageJ (version 1.53k) and GraphPad Prism v9.5.0.730.

For manuscripts utilizing custom algorithms or software that are central to the research but not yet described in published literature, software must be made available to editors and reviewers. We strongly encourage code deposition in a community repository (e.g. GitHub). See the Nature Portfolio guidelines for submitting code & software for further information.

Data

Policy information about availability of data

All manuscripts must include a data availability statement. This statement should provide the following information, where applicable:

- Accession codes, unique identifiers, or web links for publicly available datasets
- A description of any restrictions on data availability
- For clinical datasets or third party data, please ensure that the statement adheres to our policy

The cryo-EM 3D maps of the S. cerevisiae full-length Gea2 and Arl1–Gea2 complex have been deposited in the Electron Microscopy Data Bank under accession

codes EMD-28748 [https://www.ebi.ac.uk/emdb/EMD-28748] and EMD-28743 [https://www.ebi.ac.uk/emdb/EMD-28743], respectively. The consensus refined EM maps of the Gea2 and Arl1–Gea2 complex are under accession codes EMD-28749 [https://www.ebi.ac.uk/emdb/EMD-28749] and EMD-28744 [https://www.ebi.ac.uk/emdb/EMD-28744], respectively. The focused refined maps of the HDS1-3 of protomers A and B in the Arl1–Gea2 complex have accession codes of EMD-28747 [https://www.ebi.ac.uk/emdb/EMD-28747] and EMD-28746 [https://www.ebi.ac.uk/emdb/EMD-28746], respectively. The focused refined maps of the HDS1-3 domains of Gea2 protomers A and B in the Gea2 alone sample have accession codes EMD-28750 [https://www.ebi.ac.uk/emdb/EMD-28750] and EMD-28751 [https://www.ebi.ac.uk/emdb/EMD-28751], respectively. The atomic models of Gea2 and Arl1–Gea2 have been deposited in the Protein Data Bank under accession codes 8EZQ [https://doi.org/10.2210/pdb8EZQ/pdb] and 8EZJ [https://doi.org/10.2210/pdb8EZJ/pdb], respectively. The EM data are available from the authors upon reasonable request. Source data are provided with this paper. The following atomic models were used in the study from the Protein Data Bank under accession codes 7URO [https://doi.org/10.2210/pdb7URO/pdb], 5EE5 [https://doi.org/10.2210/pdb5EE5/pdb], 1UPT [https://doi.org/10.2210/pdb1UPT/pdb] and 1R8S [https://doi.org/10.2210/pdb1R8S/pdb].

Research involving human participants, their data, or biological material

Policy information about studies with human participants or human data. See also policy information about sex, gender (identity/presentation), and sexual orientation and race, ethnicity and racism.

Reporting on sex and gender	N/A
Reporting on race, ethnicity, or other socially relevant groupings	N/A
Population characteristics	N/A
Recruitment	N/A
Ethics oversight	N/A

Note that full information on the approval of the study protocol must also be provided in the manuscript.

Field-specific reporting

Please select the one below that is the best fit for your research. If you are not sure, read the appropriate sections before making your selection.

☒ Life sciences ☐ Behavioural & social sciences ☐ Ecological, evolutionary & environmental sciences

For a reference copy of the document with all sections, see nature.com/documents/nr-reporting-summary-flat.pdf

Life sciences study design

All studies must disclose on these points even when the disclosure is negative.

Sample size	The sample size for cryo-EM studies were determined by properties and qualities of the particles and also the number of particles available in each micrograph. To achieve higher resolution, at least 10,000 micrographs are routinely collected. For the current cryo-EM datasets, 20,486 raw micrographs were collected for the Arl1–Gea2 complex and 13,950 raw micrographs were collected for the Gea2 alone sample. For cellular studies, the sample size, number of replicates and choice of statistical methods were determined based on prior experience by the co-authors, literature references and common practice in the field. Three independent replicates were performed. For fluorescence microscopy, twenty cells were obtained and analyzed for each replicate.
Data exclusions	"Bad" raw particle images that did not produce 2D class averages or 3D class maps with defined features were excluded after 2D and 3D classifications. The criterion is empirical but is a standard image processing practice in the cryo-EM community.
Replication	Reproducibility resides in the large number of particles used to derive at the final 3D maps or 2D averages. The reliability and the resolution are measured by the Gold-standard Fourier shell correlation. Replication efforts with multiple refinement runs successfully yielded similar 3D maps.
Randomization	The allocation or selection of “good” and “bad” particles are determined by the computer program CryoSPARC based on the 2D templates or 3D volumes provided prior to running the program.
Blinding	The investigators cannot be blinded to the specific data points during data collection and analysis, because visual inspection is necessary to ascertain the data quality. There is no need for blinding in this type of study.

Reporting for specific materials, systems and methods

We require information from authors about some types of materials, experimental systems and methods used in many studies. Here, indicate whether each material, system or method listed is relevant to your study. If you are not sure if a list item applies to your research, read the appropriate section before selecting a response.

Materials & experimental systems

n/a	Involved in the study
☐	☒ Antibodies
☒	☐ Eukaryotic cell lines
☒	☐ Palaeontology and archaeology
☒	☐ Animals and other organisms
☒	☐ Clinical data
☒	☐ Dual use research of concern
☒	☐ Plants

Methods

n/a	Involved in the study
☒	☐ ChIP-seq
☒	☐ Flow cytometry
☒	☐ MRI-based neuroimaging

Antibodies

Antibodies used

Monoclonal ANTI-FLAG® M2 antibody produced in mouse (Catalog no: F3165-1MG, Lot SLCJ3741)
clone M2, purified immunoglobulin (Purified IgG1 subclass), 1:3500 dilution was used. MilliporeSigma (St. Louis, MO).
Anti-HA antibody produced in rabbit (Catalog no: H6908), 1:1000 dilution was used for immunoblotting. MilliporeSigma (St. Louis, MO).
Anti-Mouse IgG (H+L), HRP conjugate (W4021, 1:10,000 dilution) Lot: 0000459067 Promega (Madison, WI).
Anti-Rabbit IgG (H+L), HRP conjugate (W4011, 1:10,000 dilution) Lot: 0000529943 Promega (Madison, WI).
PGK1 Monoclonal antibody: 459250, Clone : 22C5D8, Lot: XI368684 (1:5000) Mouse / IgG1, kappa Molecular Probes Invitrogen (Eugene, OR).

Validation

Mouse Monoclonal ANTI-FLAG® M2 antibody F3165
https://www.sigmaaldrich.com/US/en/product/sigma/f3165
Google Scholar search showed that this antibody has been used in thousands of publications. One of the earliest publications using this antibody is Zhang et al. Proc Natl Acad Sci US A 1999 (PMID: 10411939), which used this antibody for immunoblotting.

Anti-HA antibody produced in rabbit: Catalog no: H6908
https://www.sigmaaldrich.com/US/en/product/sigma/h6908

PGK1 Monoclonal antibody: 459250, Clone : 22C5D8, Lot: XI368684 (1:5000) Mouse / IgG1, kappa
https://www.thermofisher.com/antibody/product/PGK1-Antibody-clone-22C5D8-Monoclonal/459250

Anti-Mouse IgG (H+L), HRP conjugate (W4021, 1:10,000) Lot: 0000459067
https://www.promega.com/products/protein-detection/primary-and-secondary-antibodies/anti_mouse-igg-h-and-l-hrp-conjugate/?catNum=W4021

Anti-Rabbit IgG (H+L), HRP conjugate (W4011, 1:10,000) Lot: 0000529943
https://www.promega.com/products/protein-detection/primary-and-secondary-antibodies/anti-rabbit-igg-h-and-l-hrp-conjugate/?catNum=W4011
